# Beyond somatotype categories: composition-based clustering of body types in young adults

**DOI:** 10.3389/fphys.2025.1722899

**Published:** 2025-11-21

**Authors:** Francesco Campa, Jordan Moon, Cristian Petri, Fabrizio Spataro, Giulia Baroncini, Eleonora Faraone, Leonardo Ortenzi, Tindaro Bongiovanni, Sofia Serafini, Pascal Izzicupo

**Affiliations:** 1 Department of Biomedical Sciences, University of Padua, Padua, Italy; 2 Body Quantification, Chicago, IL, United States; 3 Department of Sports and Computer Science, Section of Physical Education and Sports, Universidad Pablo de Olavide, Seville, Spain; 4 Section of Clinical Nutrition and Nutrigenomics, Department of Biomedicine and Prevention, University of Rome Tor Vergata, Rome, Italy; 5 Milan Lab Department, AC Milan, Milan, Italy; 6 Department of Science and Technology for Humans and the Environment, Università Campus Bio-Medico di Roma, Rome, Italy; 7 Aurelia Swimming, Roma, Italy; 8 Department of Biomedical and Neuromotor Sciences University of Bologna, Bologna, Italy; 9 Department of Medicine and Aging Sciences, “G. D’Annunzio” University of Chieti-Pescara, Chieti, Italy

**Keywords:** anthropometry, body composition, ISAK, heath and carter, kinanthropometry

## Abstract

**Background and aims:**

Somatotype analysis classifies individuals into 13 categories based on unique combinations of the three principal components: endomorphy, mesomorphy, and ectomorphy. This study aimed to examine sex-related differences and intra-category variability within somatotype classifications, and to characterize body composition patterns in the general population.

**Methods:**

Anthropometric data were collected from 185 males and 156 females aged 18–40 years to estimate somatotype, fat mass index (FMI), and skeletal muscle index (SMI). Sex differences were evaluated with Hotelling’s T^2^ and chi-square tests, while within-category morphological dispersion was quantified as Euclidean distances from centroids. K-means clustering on FMI and SMI identified Low, Medium, and High groups, and somatotype distributions across clusters were compared using chi-square and binomial tests.

**Results:**

Men exhibited an Endomorphic-Mesomorph somatotype, whereas women displayed an Mesomorph-Endomorph profile. Hotelling’s T^2^ test confirmed significant sex differences in somatotype centroids (p < 0.001), and chi-square analyses showed strong associations between sex and somatotype categories (p < 0.001). Within-category morphological dispersion was significant in most groups, with males showing greater overall variability than females (p = 0.004). K-means clustering of FMI and SMI identified Low, Medium, and High groups, with somatotype distributions differing significantly across clusters (p < 0.001); a clear predominance of a single somatotype category was observed in the Medium FMI cluster of males (Endomorphic-Mesomorph, 76.8%, p < 0.001) and in the High SMI cluster of males (Endomorphic-Mesomorph, 67.5%, p = 0.019).

**Conclusion:**

These findings highlight pronounced sex-related differences, considerable intra-category variability, and distinct body composition patterns across somatotypes in the general population. Notably, although individuals classified within the same somatotype can still present heterogeneous body shapes, the Endomorphic–Mesomorph profile distinctly characterizes males with moderate fat mass and higher muscle mass.

## Graphical Abstract

**Figure:**
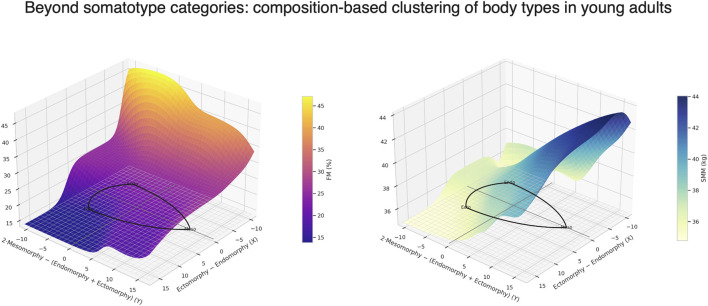

## Introduction

Kinanthropometry is the scientific discipline that applies anthropometric measurements to quantitatively and qualitatively assess body composition (Vangrunderbeek et al., 2013). Quantitative assessment refers to the direct measurement and numerical estimation of the components of body composition (e.g., mass, size and proportions of tissues), whereas qualitative assessment involves interpreting these measurements in relation to relevant outcomes such as health status, injury risk, or athletic performance (Silva, 2019). Anthropometric data are commonly used to derive simple indices, such as body mass index, which categorizes individuals based on weight divided by height squared, and waist-to-hip ratio, which assesses body fat distribution by relating waist to hip girth. Surface anthropometry also enables the monitoring of raw parameters, such as skinfold thickness, an informative marker of body fat (Campa et al., 2025), as well as their integration into predictive equations (Serafini et al., 2024), representing type-I, property-based approaches for estimating primary body mass components (Wang et al., 1995). These methods are broadly used in research, clinical practice, and sport thanks to their affordability, accessibility, and widespread adoption.

Anthropometric measurements have long been used to classify individuals based on body morphology through the concept of somatotype (Carter and Heath, 1990). This approach was first proposed by William H. Sheldon in the 1940s, linking body shape to a tripartite system based on endomorphy (relative fatness), mesomorphy (musculoskeletal robustness), and ectomorphy (linearity or leanness). The method was later refined by Heath and Carter (Carter and Heath, 1990), who developed a standardized anthropometric protocol for quantifying the three components, enabling objective, reproducible, and comparable assessments across populations. Each individual is assigned a triplet of values representing the relative levels of endomorphy, mesomorphy, and ectomorphy, which allows classification into 13 somatotype categories. These values can also be plotted on a somatochart, a two-dimensional triangular Cartesian diagram where each vertex corresponds to one of the three components. The individual’s position within the chart is mathematically determined by converting the triplet into Cartesian coordinates (X, Y), with the sum of the distances from each vertex reflecting the relative contribution of each component. This normalization constrains all possible somatotypes within a bounded 2D space, thus generating the triangular shape. As a result, the somatochart offers an intuitive visual representation of body composition, revealing the dominant component and enabling the monitoring of morphological changes over time at both individual and population levels (Pereira et al., 2017; Araújo et al., 2019).

Following puberty (approximately age 12 in females and 14 in males), rapid gains in skeletal muscle mass and strength are typically observed, driven by endocrine maturation and growth-related adaptations (Toselli et al., 2021). As individuals transition into adulthood, gradual shifts occur toward greater fat accumulation, influenced by declining basal metabolic rate, alterations in hormonal profiles, and lifestyle factors (Pataky et al., 2021; Gitsi et al., 2024). Throughout the first two decades of adulthood, skeletal muscle mass and fat mass undergo substantial remodeling (Campa et al., 2023; Campa et al., 2025). By approximately age 35, even healthy adults experience a physiological decline in skeletal muscle mass, while fat mass not only increases but also redistributes preferentially toward the abdominal region, contributing to progressive changes in overall body composition (Janssen et al., 2000; Campa et al., 2025). These trajectories are modulated by sex, habitual physical activity, and nutritional status, resulting in considerable inter-individual variability even within similar age cohorts (Janssen et al., 2025). Historically, somatotype classification has served as a practical framework for describing morphological variation across populations (Carter and Heath, 1990). However, the ability of somatotype analysis to accurately reflect underlying differences in skeletal muscle mass and fat mass remains unclear, raising the possibility that substantial heterogeneity exists within individual somatotype categories (Koleva et al., 2002; Campa et al., 2020; González Macías and Flores, 2024). Addressing this gap is essential to evaluate whether composition-based approaches, such as clustering analyses of direct body mass components, can provide a more refined characterization of body types in young adults. Integrating these methods would improve the physiological interpretation of traditional somatotype classifications by explicitly mapping distinct regions of the somatochart to specific fat–muscle distribution patterns, thereby enhancing both the accuracy and the explanatory power of somatotype-based assessments.

Therefore, the current investigation aimed to evaluate individual and sex-specific variability within the different somatotype categories and to determine whether these categories can effectively identify specific levels of skeletal muscle mass and fat mass. Such evidence could improve the interpretation and practical use of the somatotype method both at group and individual level in young adults. We hypothesize that, although individuals within the same somatotype category may vary considerably in body composition due to sex-related influences, some categories may nonetheless represent distinct clusters characterized by specific muscle and fat distribution patterns.

## Materials and methods

All research procedures were reviewed and approved by the Ethical Committee board of the University of Padova (approval code: HECDSB-022023) and conform to the Declaration of Helsinki concerning studies involving human subjects. After being provided with a detailed written explanation of the procedures, the participants gave their written informed consent.

### Participants

Recruitment took place through advertisements placed in universities, medical, recreational, market, and sports centers across Italy, running from 01/01/2025 to 01/07/2025. Exclusion criteria included the inability to collect all selected anthropometric measurements or pregnancy. A total of 341 participants aged from 18 to 40 years; 185 men (age 29.0 ± 6.0 years; body mass index = 24.2 ± 2.3 kg/m^2^) and 156 women (age 28.3 ± 7.3 years; body mass index = 21.8 ± 2.4 kg/m^2^) were involved in this study.

### Procedures

The present investigation was conceived as a multicenter, cross-sectional study. The study collected data at a national level from multiple cities across various Italian territories (Milano, Padova, Bologna, Firenze, Chieti, Pescara, and Roma). The anthropometric assessments were conducted by operators certified by the International Society for the Advancement of Kinanthropometry, following international standards (International Society for Advancement of Kinanthropometry, 2001). Body mass and height were measured using a scale with an integrated stadiometer (Seca, Hamburg, Germany), with a sensitivity of 0.1 kg and 0.1 cm, respectively. Body mass index was calculated as body mass (kg) divided by squared height (m^2^).

Skinfold thicknesses were measured using three different calipers [Harpenden, Baty International Ltd., West Sussex, United Kingdom (Original Type A caliper); Holway, California United States (Generic Type A caliper); Cescorf, Porto Alegre, Brazil (Generic Type A caliper)]. Calipers were classified following Cintra’s classification system (Cintra et al., 2025). To ensure consistency, the interclass correlation coefficient (ICC) was calculated for the three calipers based on a sample of 20 participants, yielding ICC = 0.94 (95% CI: 0.92–0.96). Girths were measured using a measuring tape (Lufkin, Apex Tool Group, United States) with a sensitivity of ±0.1 mm. Breadths were measured to the nearest 0.1 cm using a sliding caliper (Holway, California, United States).

Somatotype components were calculated according to the Heath and Carter method (Carter and Heath, 1990) as follows:

*Endomorphy* = −0.7182 + 0.1451 × X − 0.00068 × X^2^ + 0.0000014 × X^3^, where X = (sum of triceps, subscapular and supraspinal skinfolds, in mm) × (170.18/height in cm).

*Mesomorphy* = 0.858 × humerus breadth +0.601 × femur breadth +0.188 × corrected arm girth +0.161 × corrected calf girth −0.131 × height in cm + 4.5, where corrected girths = measured girth − (skinfold thickness/10).

*Ectomorphy* = if height-to-weight ratio (HWR) ≥ 40.75 = Ectomorphy = 0.732 × HWR −28.58.

If 38.25 < HWR <40.75 = Ectomorphy = 0.463 × HWR −17.63.

If HWR ≤38.25 = Ectomorphy = 0.1.

Classification was based on the relative magnitude of the three components (endomorphy, mesomorphy and ectomorphy). In this framework, “dominant” indicates that one component is at least 0.5 points higher than the other two, “similar” means that two components differ by less than or equal to 0.5 points, and “close in value” refers to cases in which all three components differ by less than or equal to 1.0 point.

### Individuals were classified into the following categories

#### Balanced Endomorphy

In this category, endomorphy is dominant, while mesomorphy and ectomorphy are similar (difference ≤0.5). Example: 4–2–2.

#### Mesomorphic-Endomorph

In this category, endomorphy is dominant and mesomorphy is higher than ectomorphy. Example: 5–3–2.

#### Mesomorph-Endomorph

In this category, endomorphy and mesomorphy are similar (difference ≤0.5) and both are higher than ectomorphy. Example: 4–4–2.

#### Endomorphic-Mesomorph

In this category, mesomorphy is dominant and endomorphy is higher than ectomorphy. Example: 3–5–1.

#### Balanced Mesomorph

In this category, mesomorphy is dominant, while endomorphy and ectomorphy are similar (difference ≤0.5). Example: 2–5–2.

#### Ectomorphic-Mesomorph

In this category, mesomorphy is dominant and ectomorphy is higher than endomorphy. Example: 2–5–3.

#### Mesomorph-Ectomorph

In this category, mesomorphy and ectomorphy are similar (difference ≤0.5) and both are higher than endomorphy. Example: 2–4–4.

#### Mesomorphic-Ectomorph

In this category, ectomorphy is dominant and mesomorphy is higher than endomorphy. Example: 2–3–4.

#### Balanced Ectomorph

In this category, ectomorphy is dominant, while endomorphy and mesomorphy are similar (difference ≤0.5). Example: 2–2–4.

#### Endomorphic-Ectomorph

In this category, ectomorphy is dominant and endomorphy is higher than mesomorphy. Example: 4–2–3.

#### Ectomorph-Endomorph

In this category, endomorphy and ectomorphy are similar (difference ≤0.5) and both are higher than mesomorphy. Example: 4–3.5–2.5.

#### Ectomorphic-Endomorph

In this category, endomorphy is dominant and ectomorphy is higher than mesomorphy. Example: 5–2–4.

#### Central

In this category, none of the three components differs by more than 1.0 point from the others. Example: 3–3–4.

Fat mass was estimated using the predictive equation proposed by Peterson et al. (2003), which requires body mass, height, age, sex, and the sum of skinfold thicknesses. Skeletal muscle mass was estimated according to the equation developed by Lee et al. (2000), based on body mass, height, age, sex, limb githrs (arm, thigh, and calf), and corresponding skinfold thicknesses. The fat mass index (FMI) and skeletal muscle index (SMI) were calculated by dividing fat mass (kg) and skeletal muscle mass (kg), respectively, by height squared (m^2^).

### Statistical analysis

All statistical analyses were performed in Python (version 3.13.7; Python Software Foundation). Somatotype components (endomorphy, mesomorphy, ectomorphy) were converted into Cartesian coordinates (X,Y) according to the Heath–Carter method (Carter and Heath, 1990), allowing the projection of individuals on the somatochart. Sex differences in mean somatotype position (X,Y) were assessed using Hotelling’s T^2^ test. Chi-square tests of independence were used to examine associations between sex and somatotype categories. Within-category morphological dispersion was quantified as the Euclidean distance of each individual from the centroid of their somatotype category (computed separately by sex). Category-level variances of these distances were calculated, and chi-square tests were used to assess whether they were significantly greater than zero. An independent samples t-test was used to compare overall dispersion between sexes. To identify groups of individuals with similar levels of body composition, separate k-means clustering analyses (k = 3, Hartigan–Wong algorithm, n_init = 50, random_state = 42) were applied to FMI and SMI values in male and female individuals. Cluster labels were reordered according to their mean values to obtain Low, Medium, and High clusters. Somatotype category distributions were compared across the three clusters using chi-square tests of independence (13 categories×3 clusters). In addition, to assess whether any single somatotype category was predominantly represented within each cluster, a *post hoc* analysis was conducted using one-tailed binomial tests (H_0_: p = 0.5). For each cluster, the most frequent category was compared against the 50% threshold. For each cluster, descriptive statistics (mean ± SD) were computed for FMI or SMI, and the relative distribution (n and %) of somatotype categories was tabulated and visualized using 100%-stacked bar plots. A significance level of p < 0.05 was adopted for all statistical tests.

## Results

The descriptive data of the participants are reported in Table 1.

**TABLE 1:** Descriptive characteristics of the participants reported as mean ± standard deviation.

Variable	Men (N = 185)	Women (N = 156)
Age (years)	29.0 ± 6.0	28.3 ± 7.3
Height (cm)	177.1 ± 6.7	164.3 ± 6.4
Body mass (kg)	76.1 ± 9.5	59.1 ± 8.6
Body mass index (kg/m^2^)	24.2 ± 2.3	21.8 ± 2.4
Endomorphy	3.1 ± 1.2	4.1 ± 1.1
Mesomorphy	5.2 ± 1.2	3.1 ± 1.1
Ectomorphy	2.1 ± 0.9	2.4 ± 1.1
SMM (kg)	40.1 ± 4.1	26.2 ± 3
FM (kg)	20.7 ± 6.0	18.1 ± 4.9
FMI (kg/m^2^)	6.6 ± 1.9	6.7 ± 1.8
SMI (kg/m^2^)	12.8 ± 0.9	9.7 ± 0.8
FM/SMM (kg/kg)	2.1 ± 0.5	1.5 ± 0.4

Abbreviation: SMM, skeletal muscle mass; FM, fat mass; FMI, fat mass index; SMI, skeletal muscle index.

Men had an average somatotype of Endomorphic-Mesomorph, whereas women showed a Mesomorph-Endomorph somatotype. The distribution of individual points by sex within the somatochart is shown in Supplementary Figure S1. A Hotelling’s T^2^ test revealed a significant difference between the two centroids (T^2^ = 203.4, F = 101.4, Mahalanobis distance = 1.5, p < 0.001).

A chi-square test of independence revealed a significant association between sex and somatotype categories (p < 0.001). The distribution of somatotype categories differed markedly between male and female participants. Among males, the most frequent categories were Endomorphic-Mesomorph (41.6%) and Balanced Mesomorph (23.2%), whereas several categories were rare or absent (i.e., Balanced Endomorph: 0%, Endomorphic-Ectomorph: 0%, Ectomorphic-Endomorph: 0.5%). Conversely, females showed higher frequencies in the Mesomorph-Endomorph (21.2%), Mesomorphic-Endomorph (19.9%), and Endomorphic-Mesomorph (18.6%) categories, while categories such as Ectomorphic-Mesomorph (1.3%), Mesomorph-Ectomorph (1.3%), and Balanced Ectomorph (3.8%) were much less represented. Supplementary Tables S1, S2 show the categories for both sexes along with the descriptive data for each of them.

**FIGURE 1:**
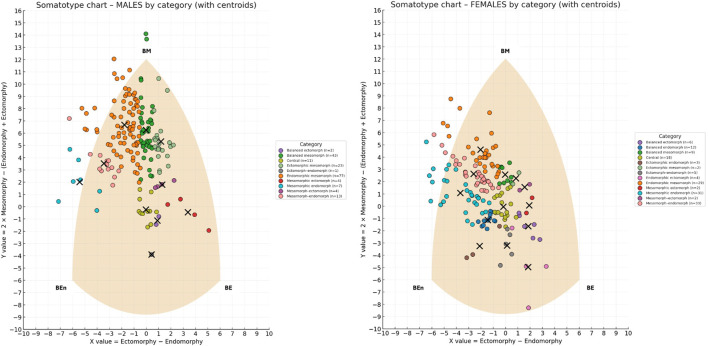
Somatotype distribution of males (left) and females (right) on the somatochart, classified by somatotype categories. Each dot represents an individual, color-coded by category, while black crosses indicate the centroid of each category. The shaded area represents the somatochart reference frame. BE (Balanced Ectomorph), BEn (Balanced Endomorph), BM (Balanced Mesomorph).

Figure 1 shows the individual points on the somatochart grouped by categories along with the centroid for each category.

When examining the within-category morphological dispersion, most categories showed a statistically significant spread of individuals around their centroid (p < 0.05), indicating that they represented heterogeneous rather than compact clusters. In males, significant dispersion was detected in several categories, including Balanced Mesomorph (mean distance = 1.8, variance = 2.9), Endomorphic–Mesomorph (mean = 2.1, variance = 1.5), Mesomorphic–Endomorph (mean = 1.9, variance = 1.3), Mesomorph–Endomorph (mean = 1.7, variance = 1.3), and Ectomorphic–Mesomorph (mean = 1.3, variance = 1.5). Additional categories such as Mesomorphic–Ectomorph (mean = 0.8, variance = 0.5), Mesomorph–Ectomorph (mean = 0.5, variance = 0.1), and Central (mean = 0.5, variance = 0.2) also exhibited significantly non-zero variance. Only Balanced Ectomorph (n = 2) and Ectomorph–Endomorph (n = 1) did not reach significance (p > 0.05).

In females, a similar pattern emerged, with significant dispersion in Endomorphic–Mesomorph (mean = 1.7, variance = 1.3), Mesomorphic–Endomorph (mean = 1.9, variance = 1.1), Mesomorph–Endomorph (mean = 1.4, variance = 0.8), and Endomorphic–Ectomorph (mean = 2.1, variance = 2.4), as well as in Balanced Mesomorph (mean = 0.5, variance = 0.1), Balanced Endomorph (mean = 0.2, variance = 0.1), Balanced Ectomorph (mean = 0.4, variance = 0.2), and Central (mean = 0.3, variance = 0.1). Significant dispersion was also found in Ectomorph–Endomorph (mean = 0.7, variance = 0.3) and Ectomorphic–Endomorph (mean = 0.7, variance = 0.5). Conversely, no significant dispersion was observed in Mesomorph–Ectomorph, Mesomorphic–Ectomorph, or Ectomorphic–Mesomorph, all of which included only two subjects. Among males, the mean distance from the category centroid was 1.7, whereas among females it was 1.3. An independent-samples t-test revealed that males showed significantly greater overall dispersion than females (t = 2.8, p = 0.004).

**FIGURE 2:**
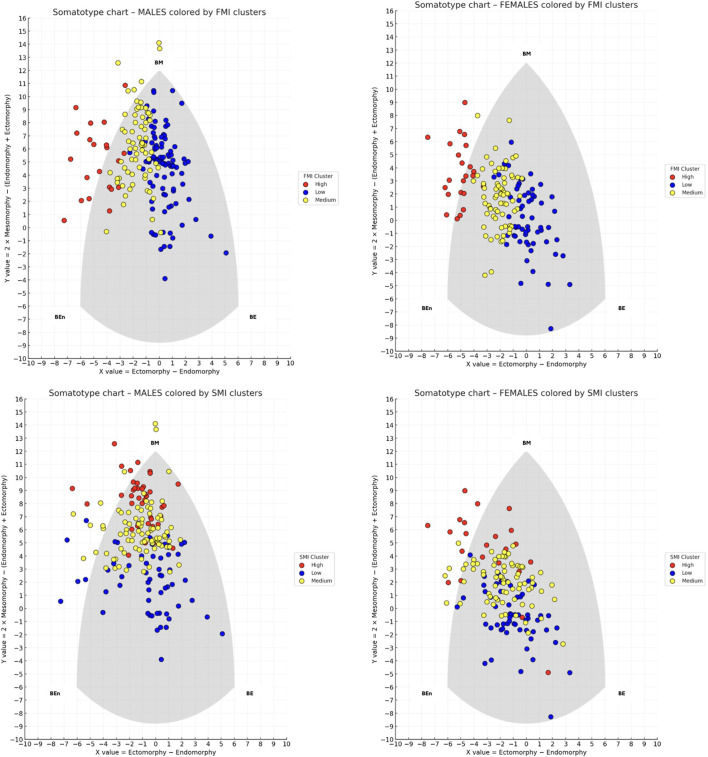
Somatotype distribution of males (left panels) and females (right panels) on the somatochart, colored by k-means clusters based on fat mass index (FMI) on the top and skeletal muscle index (SMI) on the bottom. Each dot represents an individual, with colors indicating Low (blue), Medium (yellow), and High (red) clusters. BE (Balanced Ectomorph), BEn (Balanced Endomorph), BM (Balanced Mesomorph).

For each sex and for both FMI and SMI, participants were classified into three groups (Low, Medium, High) using a k-means clustering algorithm (k = 3) applied to the univariate distributions. Figure 2 shows the results of the clustering analyses.

Regarding FMI in males, the cluster centroids were 5.2, 7.1, and 10.6, with separation thresholds at 6.2 and 8.9 FMI units. Cluster sizes and dispersion were as follows: the Low cluster included 95 participants with a mean FMI of 5.2 (±0.7); the Medium cluster included 69 participants with a mean FMI of 7.2 (±0.7); and the High cluster included 21 participants with a mean FMI of 10.6 (±1.5). A chi-square test of independence revealed a significant association between somatotype categories and FMI-based clusters (χ^2^ = 153.5, p < 0.001), indicating that the distribution of somatotype categories differed across the three clusters. Within the Medium cluster (n = 69), Endomorphic-Mesomorph emerged as clearly predominant, being observed in 53 participants (76.8%), which was significantly higher than chance (binomial test, p < 0.001). In contrast, in the Low cluster (n = 95) the most frequent category was Balanced Mesomorph (38 participants; 40.0%), and in the High cluster (n = 21) the most frequent category was Endomorphic-Mesomorph (10 participants; 47.6%), but in both cases their frequencies did not differ significantly from the 50% threshold (binomial test, p = 0.983 and p = 0.676, respectively).

**FIGURE 3:**
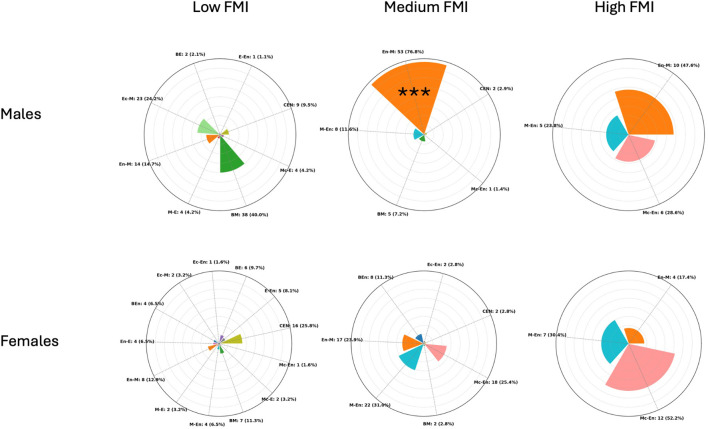
Radial plots showing the distribution of participants across somatotype categories within Low, Medium, and High fat mass index (FMI) (kg/m^2^) clusters in males (top) and females (bottom). Each bar represents the proportion (in %) of individuals within a cluster, while absolute counts with percentages are reported as external labels. The plots are scaled to a maximum radius of 80% to improve readability. CEN (Central), BE (Balanced Ectomorph), BEn (Balanced Endomorph), BM (Balanced Mesomorph), E-En (Ectomorph–endomorph), Ec-En (Ectomorphic–Endomorph), Ec-M (Ectomorphic–Mesomorph), En-E (Endomorphic–Ectomorph), En-M (Endomorphic–Mesomorph), M-E (Mesomorph–Ectomorph), Mc-E (Mesomorphic–Ectomorph), M-En (Mesomoph–Endomorph), and Mc-En (Mesomorphic–Endomorph). *** = p < 0.001.

Regarding FMI clusters in females, the centroids were 5.1, 7.0, and 10.0, with separation thresholds at 6.0 and 8.5 FMI units. Cluster sizes and dispersion were as follows: the Low cluster included 62 participants with a mean FMI of 5.0 (±0.6); the Medium cluster included 71 participants with a mean FMI of 7.07 (±0.6); and the High cluster included 23 participants with a mean FMI of 10.03 (±1.0). A chi-square test of independence revealed a significant association between somatotype categories and FMI-based clusters (χ^2^ = 95.8, p < 0.001), indicating that the distribution of somatotype categories differed across the three clusters. However, binomial tests comparing the most represented category in each cluster against a 50% threshold showed no significant predominance: Central in the Low cluster (p = 0.999), Mesomorph-Endomorph in the Medium cluster (p = 0.999), and Mesomorphic-Endomorph in the High cluster (p = 0.500). This indicates that all clusters were composed of mixed somatotype profiles, and no single category reached a statistically significant majority within any cluster. Figure 3 shows the distribution of frequencies among the FMI clusters for male and female participants.

Regarding SMI in males, the cluster centroids were 11.7, 12.8, and 14.1, with separation thresholds at 12.3 and 13.5 SMI units. Cluster sizes and dispersion were as follows: the Low cluster included 58 participants with a mean SMI of 11.71 (±0.4); the Medium cluster included 87 participants with a mean SMI of 12.89 (±0.3); and the High cluster included 40 participants with a mean SMI of 14.1 (±0.4). A chi-square test of independence revealed a significant association between somatotype categories and SMI-based clusters (χ^2^ = 83.6, p < 0.001), indicating that the distribution of somatotype categories differed across the three clusters. Binomial tests comparing the most represented category in each cluster against a 50% threshold showed that only the High cluster exhibited a clear predominance: Endomorphic-Mesomorph accounted for 67.5% (p = 0.019). In contrast, Central in the Low cluster (19.0%, p = 0.999) and Endomorphic-Mesomorph in the Medium cluster (48.3%, p = 0.666) did not reach statistical significance.

**FIGURE 4:**
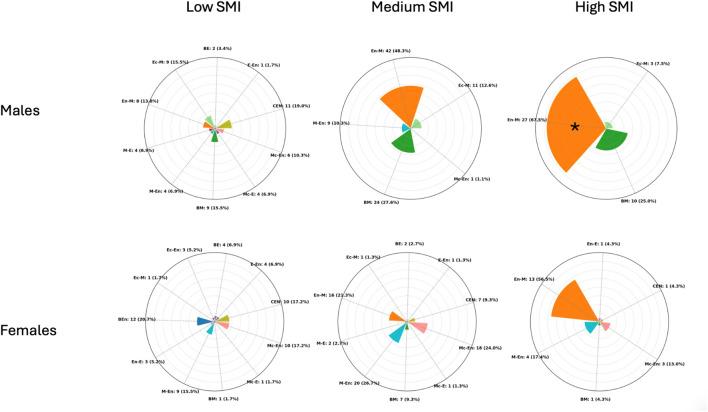
Radial plots showing the distribution of participants across somatotype categories within Low, Medium, and High skeletal muscle index (SMI) (kg/m^2^) clusters in males (top) and females (bottom). Each bar represents the proportion (in %) of individuals within a cluster, while absolute counts with percentages are reported as external labels. The plots are scaled to a maximum radius of 80% to improve readability. CEN (Central), BE (Balanced Ectomorph), BEn (Balanced Endomorph), BM (Balanced Mesomorph), E-En (Ectomorph–Endomorph), Ec-En (Ectomorphic–Endomorph), Ec-M (Ectomorphic–Mesomorph), En-E (Endomorphic–Ectomorph), En-M (Endomorphic–Mesomorph), M-E (Mesomorph–Ectomorph), Mc-E (Mesomorphic–Ectomorph), M-En (Mesomoph–Endomorph), and Mc-En (Mesomorphic–Endomorph). * = p < 0.05.

Regarding SMI in females, the cluster centroids were 8.9, 9.9, and 11.1, with separation thresholds at 9.4 and 10.5 SMI units. Cluster sizes and dispersion were as follows: the Low cluster included 58 participants with a mean SMI of 8.9 (±0.4); the Medium cluster included 75 participants with a mean SMI of 9.9 (±0.3); and the High cluster included 23 participants with a mean SMI of 11.1 (±0.6). A chi-square test of independence revealed a significant association between somatotype categories and SMI-based clusters (χ^2^ = 77.3, p < 0.001) indicating that the distribution of somatotype categories differed across the three clusters. Binomial tests comparing the most represented category in each cluster against a 50% threshold showed that no cluster exhibited a statistically significant predominance: Figure 4 shows the distribution of frequencies among the SMI clusters for male and female participants.

**FIGURE 5:**
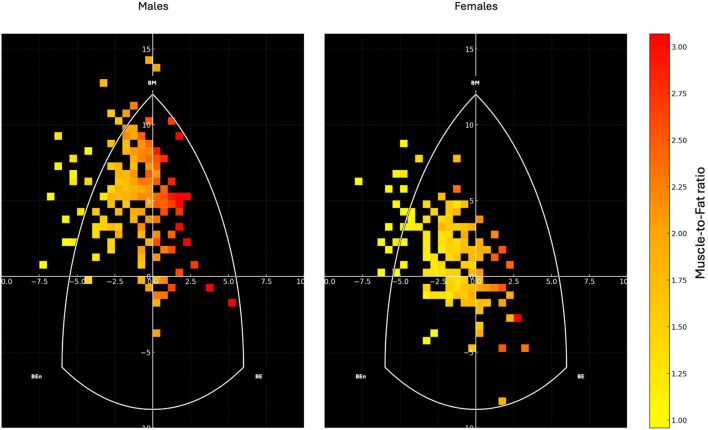
Heatmap representation of the somatotype distribution for males (left) and females (right) on the somatochart. Each square represents the density of participants within that region, color-coded according to their muscle-to-fat ratio, with warmer colors (yellow to red) indicating higher values. The white triangular boundary delineates the somatochart axes: BE (Balanced Ectomorph), BEn (Balanced Endomorph), BM (Balanced Mesomorph). The muscle-to-fat ratio was calculated as the ratio between skeletal muscle mass (kg) and fat mass (kg).

Figure 5 depicts the distribution of participants’ somatotypes based on muscle-to-fat ratio, calculated as the ratio between skeletal muscle mass (kg) and fat mass (kg).

## Discussion

The present study explored sex-specific differences and intra-category variability in somatotype distributions among young adults, and investigated whether traditional somatotype categories reflect consistent patterns of skeletal muscle and fat mass. The findings confirmed marked sexual dimorphism in body shape and composition but also revealed substantial heterogeneity within most somatotype categories, highlighting the limitations of purely morphology-based classification systems. Notably, only one category, Endomorphic-Mesomorph in males, consistently identified individuals with moderate FMI and elevated SMI. Therefore, the somatotype graphical representation should be considered primarily as a supportive tool for identifying variations in body composition, which appear to follow a gradient within the somatochart: regions located farther from the center and toward the left generally represented lower muscle-to-fat ratios, whereas regions shifting toward the right corresponded to progressively higher muscle-to-fat ratios.

Males predominantly exhibited an Endomorphic-Mesomorph profile, while females were mainly distributed in Mesomorph-Endomorph and Mesomorphic-Endomorph categories. This pattern aligns with established sex-related differences in body composition (Schorr et al., 2018), with men presenting higher skeletal muscle mass and women displaying greater relative fat mass (Campa et al., 2023; Campa et al., 2025). The clear separation between male and female centroids on the somatochart underscored the strong influence of sex on overall morphological configuration. However, the Euclidean dispersion analysis showed that individuals within the same somatotype category often diverged markedly from their group centroid. This was especially evident in males, who exhibited significantly greater morphological variability than females. Such variability indicates that while the somatotype framework is useful for visualizing population-level trends, it may obscure underlying differences in body composition at the individual level.

Somatotype does not directly quantify body compartments and, as shown in the present study, cannot capture differences in muscle or fat mass when morphology is similar (Carter and Heath, 1990). To address this limitation, individuals were clustered based on FMI and SMI. Only a few clusters displayed a predominance of a single somatotype category. Notably, in males, the Medium FMI cluster and the High SMI cluster were both dominated by the Endomorphic-Mesomorph type (76.8% and 67.5%, respectively). Interestingly, this profile is frequently observed among rugby players, particularly in defensive roles, who require high levels of muscle mass to generate force and moderate amounts of fat mass to attenuate repeated impacts during contact (Campa et al., 2020; Holway et al., 2024). Their placement within the endomorphic-mesomorph region reflects this functional balance between strength and protective mass. This indicates that while most somatotype categories encompassed individuals with heterogeneous body composition profiles, the Endomorphic-Mesomorph category in males uniquely identified individuals with moderate fat mass and high skeletal muscle mass.

Taken together, these results suggest that traditional somatotype categories only partially captured the distribution of body composition in young adults and that their interpretation should be nuanced by sex and individual body composition metrics. While the categorical framework describes general morphological trends (Hammami et al., 2018; Toselli et al., 2021), it overlooks the substantial intra-category variability revealed in this study. In this regard, the somatotype graphical representation offers a valuable complementary perspective: by plotting individuals on the somatochart, it becomes possible to visualize the continuous gradient of muscle-to-fat ratio underlying the categorical boundaries. As shown in Figure 5, regions located farther from the center and toward the left were mainly occupied by individuals with lower muscle-to-fat ratios, whereas areas toward the right and lower-right corresponded to progressively higher ratios. This spatial distribution highlights that somatotype should not be interpreted as discrete and homogeneous groups, but as positions along a continuum of body composition. The integration of composition-based clustering with the somatochart representation therefore enhances the interpretability of somatotype assessments, allowing morphological patterns to be directly mapped onto specific fat–muscle profiles.

From a practical standpoint, this graphical approach also provides an intuitive framework for monitoring individual changes in body composition over time. For instance, an individual aiming to lose weight should ideally shift their position on the somatochart from the left (lower muscle-to-fat ratio) toward the right (higher ratio). This transition must be carefully managed, as unmonitored dietary interventions may induce concurrent losses of skeletal muscle and fat mass, resulting in minimal net displacement on the somatochart or even regression toward lower muscle-to-fat ratios. However, evidence in the general population remains scarce (Vaquero-Cristóbal et al., 2015; Vaquero-Cristóbal et al., 2016), as most studies applying somatotype have focused on athletes, either to track growth or training-induced changes, or to characterize the morphological demands of different sports (Hammami et al., 2018; Toselli et al., 2021; Díaz-Martínez et al., 2024; Martínez-Mireles et al., 2025), yet they consistently show similar patterns: shifts toward the upper-left areas of the somatochart reflect increases in musculoskeletal components, whereas movements to the right indicate reductions in body fat. Athletes from sports with lower impact and greater emphasis on speed, agility, or jump performance, such as volleyball or soccer, are generally not found in the endomorphic-mesomorph region (Toselli and Campa, 2018; Bernal-Orozco et al., 2020; Petri et al., 2024). Instead, they are typically distributed along a continuum extending from the central area toward the lower-right apex of the somatochart, encompassing categories such as Central, Balanced Mesomorph, Ectomorphic-Mesomorph, Mesomorph-Ectomorph, Mesomorphic-Ectomorph, and Balanced Ectomorph. These categories are characterized by progressively lower fat mass and higher relative muscle mass, reflecting the functional demands of maintaining high power-to-weight ratios and minimizing excess non-functional mass. Although most somatotype studies have focused on athletes, these patterns provide a useful reference framework for interpreting changes also in the general population. In non-athletic individuals, similar transitions toward the upper-left areas of the somatochart, reflecting increases in musculoskeletal mass and reductions in adiposity, can indicate positive adaptations to structured exercise or improved nutritional strategies. However, unlike trained athletes, whose somatotype distribution reflects long-term morphological specialization to sport-specific demands (e.g., enhanced mesomorphy in strength and power sports or greater ectomorphy in endurance athletes), individuals from the general population display more heterogeneous and less polarized profiles. Thus, understanding the athlete-based patterns allows contextualizing the direction and magnitude of somatotype shifts in normal subjects: for instance, movement toward a more mesomorphic-ectomorphic configuration may suggest an improvement in body composition and physical performance, even if absolute levels of muscularity remain lower than in trained populations. This sport-specific patterns illustrate how the somatochart can contextualize individual morphological profiles according to the physiological requirements of different athletic disciplines. Therefore, interpreting longitudinal shifts within the somatochart should always consider both fat and muscle components to ensure that improvements in body composition reflect true gains in relative muscle mass rather than non-specific reductions in overall body mass.

This study has some limitations. First, the results cannot be generalized beyond the examined population of young adults aged 18–40 years. Different age groups or populations may display distinct somatotype distributions and body composition characteristics, and further studies are needed to verify whether the present findings apply to other cohorts. Second, the estimates of fat mass and skeletal muscle mass reported in this study are representative of the specific reference methods used to develop the prediction equations adopted. In particular, the Peterson equation (Peterson et al., 2003) estimates fat mass based on a four-compartment model, which may yield values that differ from those obtained using dual-energy X-ray absorptiometry, a more commonly employed reference method. Conversely, the Lee equation (Lee et al., 2000) used to estimate skeletal muscle mass is less concerning in this respect, as current anthropometric models for skeletal muscle mass have been developed and validated exclusively against magnetic resonance imaging, which remains the gold standard reference technique. In addition, SMI can be calculated from different muscle-related parameters, most commonly skeletal muscle mass or appendicular lean soft mass. Because these two approaches do not yield interchangeable values, the use of one method over the other may lead to substantially different SMI estimates (Paoli and Campa, 2024). In this study, SMI was calculated as SMM divided by height squared; therefore, comparisons should only be made with studies adopting an SMM-based procedure.

## Conclusion

This study demonstrated that somatotype classification, while revealing clear sex-related patterns, encompassed considerable within-category heterogeneity, especially among males. Most categories included individuals with diverse combinations of fat and muscle mass, highlighting the limited ability of somatotype alone to accurately represent body composition. However, the Endomorphic-Mesomorph category in males consistently identified individuals with high skeletal muscle mass and moderate fat mass, suggesting that some somatotype groups may reflect distinctive composition profiles. Rather than serving as a standalone classification tool, somatotype should therefore be interpreted primarily through its graphical representation on the somatochart. This visual approach enables the observation of progressive gradients in body composition: individuals positioned farther to the left tended to show lower muscle-to-fat ratios, whereas those positioned more toward the right tended to show higher ratios. By locating individuals along this continuum, the somatochart can support a more physiologically meaningful interpretation of morphological patterns, enabling the monitoring of compositional changes over time and offering practical insights for research, clinical, and sports contexts.

## Data Availability

The raw data supporting the conclusions of this article will be made available by the authors, without undue reservation.

## Supplementary material

SUPPLEMENTARY FIGURE S1 On the left, a somatochart is shown with the main coordinates used to define the 13 somatotype categories. On the right, individual somatotypes of male (blue) and female (green) participants are plotted, along with their respective centroids. CEN (Central), BE (Balanced Ectomorph), BEn (Balanced Endomorph), BM (Balanced Mesomorph), E-En (Ectomorph–Endomorph), Ec-En (Ectomorphic–Endomorph), Ec-M (Ectomorphic–Mesomorph), En-E (Endomorphic–Ectomorph), En-M (Endomorphic–Mesomorph), M-E (Mesomorph–Ectomorph), Mc-E (Mesomorphic–Ectomorph), M-En (Mesomoph–Endomorph), and Mc-En (Mesomorphic–Endomorph).
