# Supplementary material for: Beyond somatotype categories: composition-based clustering of body types in young adults
Source: Front Physiol. 2025 Nov 21;16:1722899. doi: 10.3389/fphys.2025.1722899 (PMC12679298; doi:10.3389/fphys.2025.1722899)
Supplement: Supplementary file 1 [file Table1.docx]

| **Supplementary table 1. Descriptive characteristics of the male participants grouped by somatotype categories.** | | | | | | | | | | |
| --- | --- | --- | --- | --- | --- | --- | --- | --- | --- | --- |
| Variable | Central | Ectomorph-Endomorph | Balanced Ectomorph | Ectomorphic-Mesomorph | Endomorphic-Mesomorph | Mesomorph-Ectomorph | Mesomorph-Endomorph | Balanced Mesomorph | Mesomorphic-Ectomorph | Mesomorphic-Endomorph |
| Age (years) | 28.8 ± 6.7 | 29.0 ± nan | 30.0 ± 4.2 | 30.4 ± 6.7 | 28.1 ± 6.4 | 26.5 ± 6.9 | 29.7 ± 5.6 | 29.7 ± 7.2 | 23.8 ± 3.5 | 32.9 ± 4.3 |
| Stature (cm) | 184.2 ± 6.1 | 180.0 ± nan | 175.2 ± 6.0 | 175.4 ± 7.3 | 175.9 ± 6.1 | 177.8 ± 3.4 | 175.9 ± 8.3 | 179.0 ± 6.0 | 184.5 ± 1.3 | 171.6 ± 4.3 |
| Body mass (kg) | 76.1 ± 7.1 | 69.7 ± nan | 62.4 ± 4.6 | 68.1 ± 9.1 | 78.7 ± 9.0 | 65.5 ± 3.4 | 79.3 ± 9.6 | 76.5 ± 8.1 | 65.4 ± 5.4 | 81.7 ± 7.7 |
| Endomorphy | 3.3 ± 0.6 | 3.0 ± nan | 2.9 ± 0.5 | 1.7 ± 0.4 | 3.3 ± 0.8 | 2.4 ± 0.6 | 4.9 ± 0.7 | 2.4 ± 0.4 | 1.6 ± 0.6 | 5.9 ± 1.1 |
| Mesomorphy | 3.2 ± 0.7 | 1.3 ± nan | 2.7 ± 0.2 | 5.0 ± 0.8 | 5.8 ± 0.8 | 4.0 ± 0.2 | 4.9 ± 0.7 | 5.5 ± 1.0 | 3.1 ± 0.5 | 4.2 ± 1.2 |
| Ectomorphy | 3.3 ± 0.2 | 3.4 ± nan | 3.8 ± 0.3 | 2.9 ± 0.4 | 1.5 ± 0.7 | 3.7 ± 0.2 | 1.4 ± 0.5 | 2.3 ± 0.4 | 5.0 ± 0.9 | 0.5 ± 0.3 |
| SMM (kg) | 38.4 ± 3.1 | 36.7 ± nan | 34.9 ± 0.6 | 38.7 ± 4.2 | 41.1 ± 3.8 | 37.5 ± 1.6 | 38.8 ± 4.0 | 41.7 ± 4.0 | 37.8 ± 2.2 | 34.8 ± 3.2 |
| FM (kg) | 19.9 ± 3.4 | 17.9 ± nan | 14.2 ± 1.6 | 14.5 ± 2.8 | 22.8 ± 5.1 | 14.8 ± 3.0 | 26.5 ± 4.4 | 18.3 ± 2.2 | 11.6 ± 3.2 | 33.0 ± 7.4 |
| FMI (kg/m2) | 5.8 ± 0.7 | 5.5 ± nan | 4.6 ± 0.2 | 4.7 ± 0.6 | 7.4 ± 1.5 | 4.7 ± 0.9 | 8.5 ± 1.2 | 5.7 ± 0.5 | 3.4 ± 1.0 | 11.1 ± 2.1 |
| SMI (kg/m2) | 11.3 ± 0.5 | 11.3 ± nan | 11.4 ± 0.6 | 12.5 ± 0.7 | 13.3 ± 0.8 | 11.9 ± 0.2 | 12.5 ± 0.6 | 13.0 ± 0.7 | 11.1 ± 0.5 | 11.8 ± 0.5 |
| FM/SMM (kg/kg) | 2.0 ± 0.2 | 2.1 ± nan | 2.5 ± 0.2 | 2.7 ± 0.4 | 1.9 ± 0.3 | 2.6 ± 0.6 | 1.5 ± 0.2 | 2.3 ± 0.2 | 3.4 ± 0.9 | 1.1 ± 0.2 |
| Abbreviation: SMM, skeletal muscle mass; FM, fat mass; FMI, fat mass index; SMI, skeletal muscle index. | | | | | | | | | | |
