# Supplementary material for: Beyond somatotype categories: composition-based clustering of body types in young adults
Source: Front Physiol. 2025 Nov 21;16:1722899. doi: 10.3389/fphys.2025.1722899 (PMC12679298; doi:10.3389/fphys.2025.1722899)
Supplement: Supplementary file 2 [file Table2.docx]

| **Supplementary table 2**. Descriptive characteristics of the female participants grouped by somatotype categories. | | | | | | | | | | | | | |
| --- | --- | --- | --- | --- | --- | --- | --- | --- | --- | --- | --- | --- | --- |
| Variable | Central | Ectomorph-Endomorph | Balanced Ectomorph | Ectomorphic-Endomorph | Ectomorphic-Mesomorph | Balanced Endomorph | Endomorphic-Ectomorph | Endomorphic-Mesomorph | Mesomorph-Ectomorph | Mesomorph-Endomorph | Balanced Mesomorph | Mesomorphic-Ectomorph | Mesomorphic-Endomorph |
| Age (years) | 27.7 ± 8.1 | 26.4 ± 7.8 | 30.2 ± 7.1 | 30.7 ± 9.5 | 23.0 ± 1.4 | 29.5 ± 8.4 | 27.2 ± 6.2 | 28.0 ± 7.9 | 21.0 ± 0.0 | 27.0 ± 6.8 | 31.3 ± 5.7 | 25.5 ± 4.9 | 30.0 ± 7.0 |
| Stature (cm) | 163.9 ± 5.0 | 166.9 ± 7.4 | 170.8 ± 3.5 | 169.6 ± 6.2 | 164.6 ± 11.4 | 164.5 ± 5.8 | 174.2 ± 9.9 | 164.0 ± 7.4 | 156.9 ± 8.9 | 162.0 ± 5.1 | 163.6 ± 8.5 | 161.8 ± 4.5 | 164.5 ± 4.4 |
| Body mass (kg) | 53.3 ± 5.3 | 53.9 ± 9.2 | 54.5 ± 4.5 | 61.0 ± 9.6 | 56.7 ± 13.9 | 55.7 ± 6.2 | 56.4 ± 10.6 | 63.2 ± 10.2 | 46.6 ± 7.4 | 59.5 ± 7.2 | 56.4 ± 9.1 | 49.6 ± 4.7 | 63.8 ± 6.7 |
| Endomorphy | 3.5 ± 0.5 | 3.8 ± 0.6 | 2.6 ± 0.8 | 5.2 ± 0.8 | 2.1 ± 0.8 | 4.4 ± 0.4 | 2.9 ± 0.7 | 3.7 ± 0.8 | 1.8 ± 0.4 | 4.5 ± 0.7 | 2.8 ± 0.4 | 1.7 ± 0.2 | 5.3 ± 0.8 |
| Mesomorphy | 3.4 ± 0.4 | 2.2 ± 0.7 | 2.7 ± 0.9 | 2.5 ± 0.6 | 3.6 ± 0.3 | 3.2 ± 0.3 | 1.4 ± 0.9 | 5.0 ± 0.8 | 3.4 ± 0.0 | 4.5 ± 0.6 | 4.0 ± 0.4 | 2.7 ± 0.4 | 4.0 ± 0.7 |
| Ectomorphy | 3.3 ± 0.5 | 3.9 ± 0.6 | 4.4 ± 0.4 | 3.0 ± 0.6 | 2.9 ± 0.4 | 3.0 ± 0.4 | 4.8 ± 1.1 | 1.7 ± 0.8 | 3.4 ± 0.1 | 1.9 ± 0.8 | 2.7 ± 0.3 | 3.7 ± 0.1 | 1.6 ± 0.9 |
| SMM (kg) | 25.1 ± 2.4 | 24.5 ± 2.6 | 27.1 ± 2.9 | 24.0 ± 0.7 | 26.9 ± 5.6 | 24.4 ± 1.9 | 26.9 ± 6.5 | 28.6 ± 3.9 | 24.1 ± 2.8 | 25.8 ± 2.5 | 26.4 ± 2.9 | 24.6 ± 1.0 | 26.2 ± 1.6 |
| FM (kg) | 14.3 ± 1.8 | 14.3 ± 1.9 | 12.3 ± 1.7 | 20.2 ± 5.5 | 13.2 ± 1.5 | 17.5 ± 2.8 | 12.9 ± 1.3 | 18.8 ± 4.5 | 9.7 ± 1.0 | 19.8 ± 3.8 | 14.5 ± 3.0 | 10.0 ± 0.1 | 22.8 ± 4.6 |
| FMI (kg/m2) | 5.3 ± 0.5 | 5.1 ± 0.3 | 4.2 ± 0.5 | 6.9 ± 1.5 | 4.9 ± 0.1 | 6.4 ± 0.7 | 4.3 ± 0.6 | 7.0 ± 1.5 | 3.9 ± 0.1 | 7.5 ± 1.3 | 5.4 ± 0.6 | 3.8 ± 0.2 | 8.4 ± 1.7 |
| SMI (kg/m2) | 9.3 ± 0.6 | 8.8 ± 0.5 | 9.3 ± 0.7 | 8.4 ± 0.7 | 9.9 ± 0.7 | 9.0 ± 0.2 | 8.8 ± 1.2 | 10.6 ± 0.9 | 9.8 ± 0.0 | 9.8 ± 0.6 | 9.8 ± 0.4 | 9.4 ± 0.2 | 9.7 ± 0.7 |
| FM/SMM (kg/kg) | 1.8 ± 0.2 | 1.7 ± 0.1 | 2.2 ± 0.5 | 1.3 ± 0.4 | 2.0 ± 0.2 | 1.4 ± 0.2 | 2.1 ± 0.4 | 1.6 ± 0.3 | 2.5 ± 0.0 | 1.3 ± 0.2 | 1.9 ± 0.2 | 2.4 ± 0.1 | 1.2 ± 0.2 |
| Abbreviation: SMM, skeletal muscle mass; FM, fat mass; FMI, fat mass index; SMI, skeletal muscle index. | | | | | | | | | | | | | |
