# Supplementary figures and images for: Beyond somatotype categories: composition-based clustering of body types in young adults
Source: Front Physiol. 2025 Nov 21;16:1722899. doi: 10.3389/fphys.2025.1722899 (PMC12679298; doi:10.3389/fphys.2025.1722899)

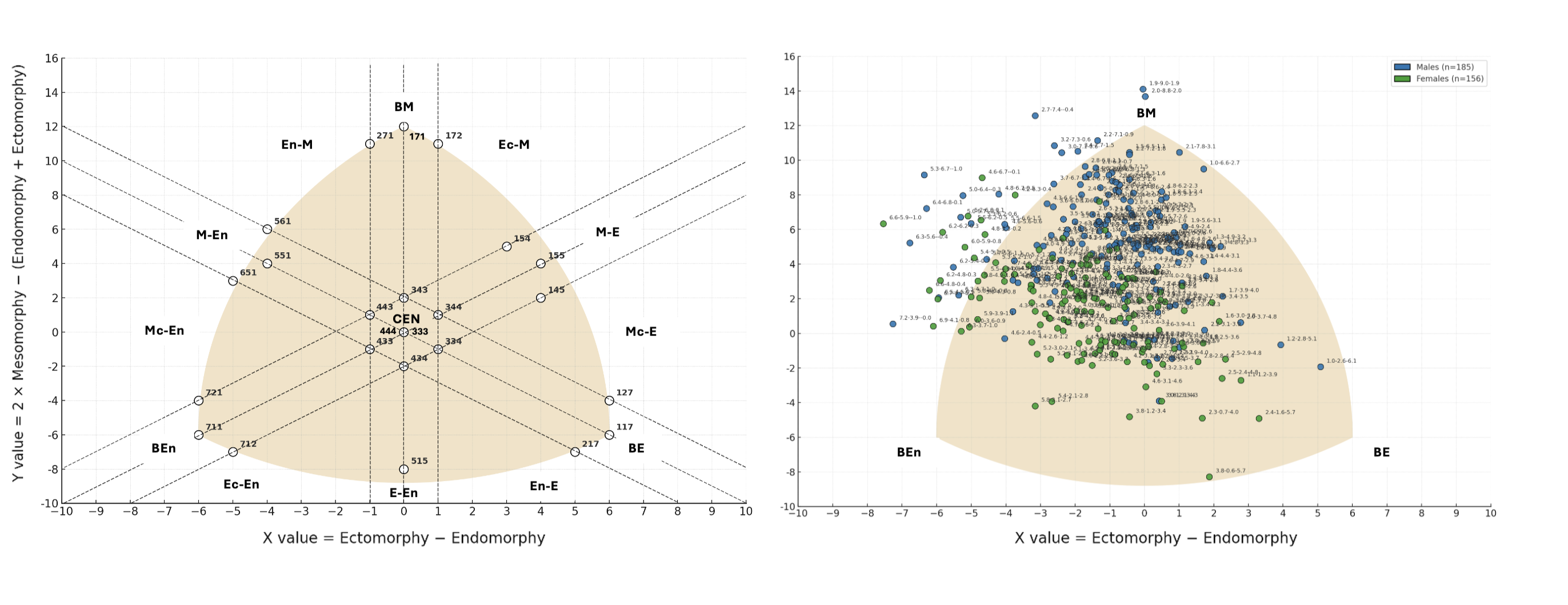

Supplement: Supplementary file 3 [file Image1.png]
